# Comparison of Phacoemulsification Alone and With Trabecular Microbypass Stent in Primary Open-Angle Glaucoma and Normal-Tension Glaucoma: An 18-Month Outcome Study

**DOI:** 10.1155/2024/4034215

**Published:** 2024-11-07

**Authors:** Yu-Ting Tsao, Po-Han Yeh, Wei-Wen Su

**Affiliations:** ^1^Department of Ophthalmology, Chang Gung Memorial Hospital, Linkou 333423, Taiwan; ^2^Department of Ophthalmology, New Taipei Municipal TuCheng Hospital, New Taipei City 236017, Taiwan; ^3^Department of Medicine, Chang Gung University College of Medicine, Taoyuan 33302, Taiwan

## Abstract

iStent (Glaukos, San Clemente, CA, USA), a minimally invasive glaucoma device, reduces intraocular pressure (IOP) by enhancing aqueous humor outflow when implanted into Schlemm's canal. Although it has demonstrated effectiveness in lowering IOP and slowing glaucoma progression, its applicability to the Taiwanese population, known for a higher incidence of normal-tension glaucoma (NTG) cases, requires validation. This retrospective case-control study, conducted from January 2018 to December 2020, aimed to assess the effectiveness of combining first-generation iStent with phacoemulsification (phaco-iStent) compared to phacoemulsification alone in Taiwanese patients diagnosed with primary open-angle glaucoma (POAG) and NTG, involving 71 eyes (iStent group: POAG 16 and NTG 8; control group: POAG 28 and NTG 19). The primary outcomes included changes in IOP and the number of antiglaucoma medications, with subgroup analyses for POAG and NTG. Over the 18-month follow-up, the iStent group achieved a significantly greater reduction in antiglaucoma medications compared with the control group (*p* value = 0.003∼<0.001) without significant IOP rebound. After adjusting for confounding factors, the reduction in the number of antiglaucoma medications with iStent remained significant (*β* = −0.8, *p* < 0.001) according to the generalized estimating equation. At 18 months, the iStent group demonstrated higher qualified and complete success rates than the control group (73.3% vs. 16.7%, *p* value = 0.001% and 53.3% vs. 0%, *p* value = 0.002, respectively). Notably, the NTG-iStent subgroup achieved the highest complete success rate (85.7% at 18 months). In conclusion, phaco-iStent emerges as an effective standalone treatment for Taiwanese patients with POAG and NTG, providing substantial IOP reduction and higher success rates, especially in NTG cases. These findings propose that phaco-iStent could be a promising intervention for managing POAG and NTG within the Taiwanese population.

**Trial Registration:** ClinicalTrials.gov identifier: NCT06630546.

## 1. Introduction

Glaucoma is one of the leading causes of irreversible vision loss and blindness globally, impacting over 50 million people [1]. In the United States, the cost of treating glaucoma patients annually surpasses $2.5 billion, which is a considerable financial burden on patients, families, and the government [2]. Topical antihypertensive ophthalmic agents are usually the first-line therapy for glaucoma. If the disease continues to progress or if the patient is intolerant to these medications, further surgical treatment should be considered. Conventional glaucoma surgery involves the creation of a drainage fistula to facilitate aqueous humor drainage, which, while effective, carries the risk of severe complications, including hypotony, choroidal effusion and hemorrhage, bleb leaks, blebitis, and bleb-related dysesthesia [3]. In recent years, various new procedures have been developed that provide a safer means of reducing IOP by enhancing the drainage of aqueous humor through the natural outflow pathways.

**Figure 1:**
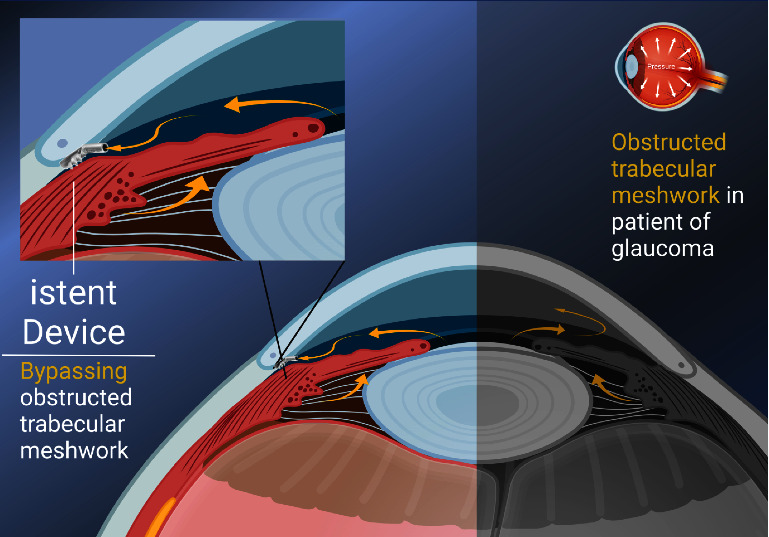
Schematic diagram illustrating the intraocular pressure reduction mechanism of iStent trabecular microbypass stent. In open-angle glaucoma, it is believed that the obstructed outflow of aqueous humor at the trabecular meshwork plays the most important role. The stagnant aqueous humor in the eye may result in ocular hypertension and further glaucoma (right side of the picture). The iStent could insert through the obstructed trabecular meshwork and bypass the physiologic aqueous humor outflow directly into the Schlemm's canal. Therefore, iStent could be used to maintain the adequate IOP and treat glaucoma (left side of the picture). Created in BioRender. Tsao (2022) https://BioRender.com/m87f352.

The iStent, developed by Glaukos Corporation in Laguna Hills, CA, USA, is a trabecular microbypass stent and the first USA FDA-approved minimally invasive glaucoma surgery (MIGS) implant. Composed of nonferromagnetic titanium, this 1 mm stent includes three retention arches that can be implanted into Schlemm's canal along with a snorkel to enhance physiological aqueous humor outflow directly into the canal [4]. The iStent can be placed using an ab interno approach, which allows for implantation with phacoemulsification to reduce the number of surgical incisions and improve safety [5]. However, due to its efficacy and safety profile, an escalating body of research supports iStent as a standalone procedure [6]. The mechanism by which iStent lowers IOP is demonstrated in Figure 1. Previous research has indicated that iStent implantation combined with phacoemulsification can reduce IOP and the number of required antiglaucoma medications over the long term. However, the effectiveness of this treatment may vary depending on the individual's race and the type of glaucoma they have [7–12].

Taiwan, like many East Asian countries, has a high incidence of normal-tension glaucoma (NTG), a type of open-angle glaucoma that is characterized by glaucomatous optic neuropathy with consistently normal IOP [13]. While the angle structure and visual impairment of NTG and primary open-angle glaucoma (POAG) may be similar, their underlying pathological mechanisms may differ [14]. Despite this, no published studies have compared the efficacy of iStent implantation in these two patient groups. Therefore, the objective of this study was to assess the safety and efficacy of phacoemulsification combined with one trabecular microbypass stent implantation (phaco-iStent) versus phacoemulsification alone in a Taiwanese population, as well as to investigate any differences in outcomes between patients with POAG and those with NTG.

## 2. Materials and Methods

### 2.1. Study Design and Population

This study was a retrospective case-control study conducted at the Department of Ophthalmology at Chang Gung Memorial Hospital, Linkou Branch. The study adhered strictly to the principles outlined in the Declaration of Helsinki and obtained approval from the institutional review board of Chang Gung Memorial Hospital in 2022 (IRB number: 202200593B0). A preprint has previously been published [15].

Between January 1, 2018, and December 31, 2020, we enrolled POAG and NTG patients who underwent phacoemulsification with or without first-generation iStent (Glaukos, San Clemente, CA, USA) implantation. The patients who underwent phacoemulsification combined with iStent implantation were assigned to the study group (iStent group), while those who received phacoemulsification alone comprised the control group. To meet the diagnostic criteria for POAG, patients had to have an untreated IOP exceeding 22 mmHg at varying times of the day, open anterior chamber angles on gonioscopy, glaucomatous optic disc cupping with a cup-to-disc ratio of 0.7 or greater, with thinning or notching of the neural rim, and characteristic optic nerve-related visual field loss on Humphrey perimetry. The diagnostic criteria for NTG were the same as those for POAG, except that the pretreatment IOP never exceeded 22 mmHg. We only included patients who had clinically significant cataracts with a best-corrected visual acuity (BCVA) of 20/40 (LogMAR 0.3) or worse. We excluded patients diagnosed with angle-closure glaucoma, uveitis, steroid responders, secondary glaucoma, amblyopia, and those who had previously undergone any glaucoma surgery or MIGS implantation. There was no medication washout period before surgery.

## 3. Devices and Surgical Techniques

In all patients, a standard phacoemulsification procedure was performed, along with the implantation of a foldable posterior chamber intraocular lens (PCIOL). In the iStent group, we employed an additional ophthalmic viscosurgical device (OVD) to fill the anterior chamber. To achieve optimal visualization of the nasal angle, the patient's head was rotated away from the surgeon at 35°, and the surgical microscope was tilted toward the surgeon at the same angle. An intraoperative gonioscope (Ocular Hill Open Access Surgical Gonio—Left Hand, OHSOG-LH, surgical gonio lens) was used to visualize the angle directly, while an additional OVD was applied to the cornea's surface. A preloaded microbypass stent (model GTS100L) was then inserted through the temporal corneal incision into the nasal Schlemm's canal. We confirmed the position of the iStent and checked for blood reflux to ensure proper placement. Following iStent implantation, we removed the OVD and sealed the corneal wound with intrastromal hydration. Adequate intraocular pressure (IOP) was assessed by palpation at the end of the surgery. Postoperative medications included a topical fluoroquinolone antibiotic four times daily and topical prednisolone acetate 1% two to four times daily for 1 month.

## 4. Data Collection

We acquired all clinical data from the electronic medical record, operation record, and examination reports. Baseline data were collected during the preoperative visit immediately before the procedure, while postoperative data were collected during the postoperative visits at 1, 2, 3, 6, 12, and 18 months after surgery. We utilized a pneumotonometer (Canon, TX-10, Canon Corporation, Tokyo, Japan) to measure IOP, Heidelberg Spectralis-OCT (Spectralis SD-OCT; Heidelberg Engineering, Heidelberg, Germany) to evaluate optic disc morphology, and Humphrey Field Analyzer (HFA; Carl Zeiss Meditec, Dublin, CA) to perform visual field examinations. This study recorded the mean deviation (MD) and visual field index (VFI) to quantify the visual field conditions. We compared the results before and 1 year after surgery.

As medication washout was not conducted before surgery in this study, we calculated the estimated washout intraocular pressure (ewIOP) by multiplying the patient's IOP by the mean percentage of IOP reduction associated with each glaucoma medication taken. These reduction percentages were obtained from Tables 1 to 7 of the BCSC 2020-2021 series: Section 10–glaucoma, in addition to providing data on IOP and medication changes [16]. The IOP weightings for each antiglaucoma drug were listed in Supporting Table 1, and the formula used was described as follows:

(1) $$\begin{array}{c} \text{ew IOP}=\text{uwIOP}\times \left(1+\text{mean IOP reduction weighting of antiglaucoma agents}\right). \end{array}$$

For example:

i. For timolol monotherapy, ewIOP = IOP × 1.25
ii. For timolol and latanoprost dual therapy, ewIOP = IOP × 1.25 × 1.285

## 5. Outcome Measurement

The primary outcome measures of this study were the percentage changes in IOP, number of glaucoma medications, ewIOP, and visual acuity (LogMAR). We also assessed the proportion of eyes that achieved surgical success. Complete success was defined as postsurgical IOP of less than 21 mmHg without the use of any glaucoma medication, while qualified success was defined as postsurgical IOP not exceeding 21 mmHg with a reduction of one or more antiglaucoma agents compared to baseline. Additionally, we conducted subgroup analysis for POAG and NTG.

### 5.1. Statistical Analyses

Statistical analyses were performed using IBM SPSS Statistics for Windows, Version 22.0. Armonk, NY: IBM Corp. Descriptive statistics were used for patient characterization, with means and standard deviations or proportions presented as appropriate. The Kolmogorov–Smirnov test was used to test the normality of the distribution for continuous variables. To compare values between the study and control groups, the Student's *t*-test was used for normally distributed data, and the Mann–Whitney *U* test was used for non-normally distributed data. Generalized estimating equations were used to evaluate the direct effect of iStent implantation on the changes in antiglaucoma medications and ewIOP after adjusting for confounding factors, such as age, eye site, sex, and disease diagnosis, and underlying diseases such as diabetes mellitus, hypertension, coronary artery disease, baseline visual acuity (LogMAR), baseline unwashed IOP, baseline ewIOP, baseline number of glaucoma medications, baseline retinal nerve fiber layer (RNFL) thickness on OCT, baseline MD, and baseline VFI. Statistical significance was defined as a *p* value less than 0.05.

## 6. Results

### 6.1. Demographics and Baseline Ocular Characteristics

The study enrolled 71 OAG patients, each contributing one eye to the analysis. Of these, 24 eyes underwent phaco-iStent, while 47 received phacoemulsification alone.  Table 1 provides demographic and clinical data for each group. In the phaco-iStent group, there were 16 patients with POAG and eight with NTG, while, in the control group, there were 28 POAG and 19 NTG patients. There were no statistically significant differences between the two groups in terms of demographic or clinical characteristics.

### 6.2. IOP, Number of Medications, and Visual Acuity

**Figure 2:**
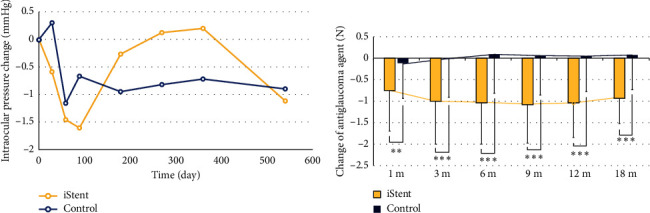
The intraocular pressure (IOP) changes and antiglaucoma medication changes in the iStent group and control group throughout the 18-month follow-up. (a) Both the iStent group and control group showed a slight decrease in IOP after the surgery. However, there is no significant difference in IOP reduction between the two groups. (b) The iStent group showed a better reduction of antiglaucoma medications than the control group throughout the 18-month follow-up. The average reduction of antiglaucoma agent was −0.75 ± 0.94 in the iStent group and −0.13 ± 0.61 in the control group at 1 month (*p* value = 0.003); −1.00 ± 0.98 in the iStent group and −0.02 ± 0.89 in the control group at 3 months (*p* value < 0.001); −1.04 ± 0.95 in the iStent group and 0.08 ± 0.14 in the control group at 6 months (*p* value < 0.001); −1.08 ± 0.88 in the iStent group and 0.05 ± 0.91 in the control group at 9 months (*p* value < 0.001); −1.05 ± 0.79 in the iStent group and 0.03 ± 0.80 in the control group at 12 months (*p* value < 0.001); and −0.93 ± 0.59 in the iStent group and 0.05 ± 0.80 in the control group at 18 months (*p* value < 0.001).

Figures 2(a) and 2(b) depict changes in IOP and number of antiglaucoma medications in the iStent and control groups. The average change in IOP ranged from −2 to +0.5 mmHg at each follow-up visit. The mean IOP reduction difference between the two groups remained consistently within a 1 mmHg range, with no statistically significant difference observed (Figure 2(a) and Supporting Table 2). However, the iStent group demonstrated a significant reduction in the average number of antiglaucoma medications used (between −0.75 and −1.08) compared to the control group (+0.08 to −0.13) (*p* value range from 0.003 to < 0.001). Despite having a similar baseline number of antiglaucoma medications, the iStent group showed a significant reduction in postoperative medication use at all time points (Figure 2(b) and Supporting Table 3). Additionally, we calculated the ewIOP, and the result showed a significant reduction in the iStent group compared to the control group (Supporting Figure 1). The decrease of ewIOP was consistently greater in the iStent group than in the control group throughout the 18-month follow-up, with statistical significance observed at 1, 3, 6, 12, and 18 months (−15.06% to −21.92% vs. +1.66% to −4.73%, *p* value 0.005∼0.045, Supporting Table 4).

To further evaluate the direct effect of iStent implantation on changes in antiglaucoma medication, we performed a generalized estimating equation analysis across all seven visits. Our results showed a significant effect of iStent implantation on the change in antiglaucoma medication usage (*β* = −0.8, SE = 0.165, *p* value < 0.001, Table 2), after adjusting for all confounding factors as listed in Table 1. These factors include age, laterality, sex, disease diagnosis, systemic diseases (such as diabetes mellitus, hypertension, and coronary artery disease), baseline visual acuity (LogMAR), baseline IOP, baseline number of antiglaucoma medications used, baseline RNFL thickness, baseline MD, and baseline VFI. Additionally, we performed a generalized estimating equation analysis to assess the correlation between iStent use and changes in ewIOP. The results indicated a significant effect of iStent implantation on the change in ewIOP (*β* = −16.083, SE = 4.099, *p* < 0.001; see Supporting Table 5).

### 6.3. Surgical Success

**Figure 3:**
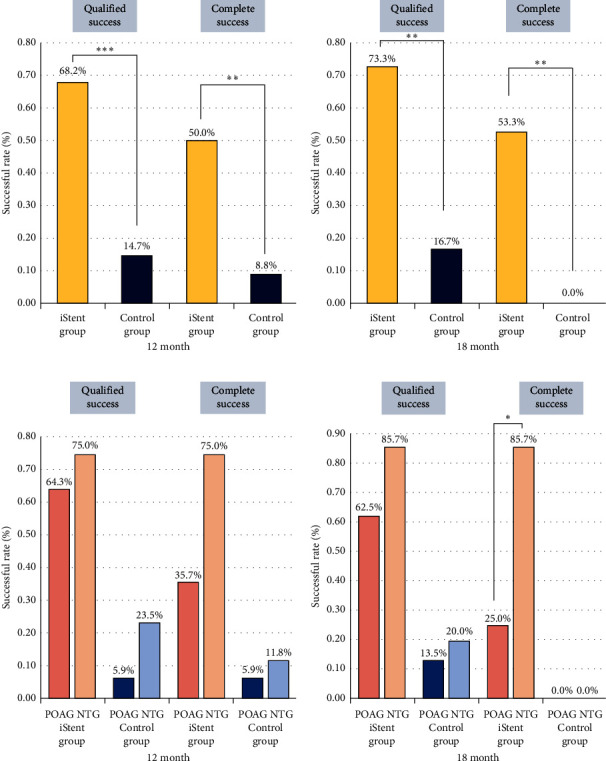
The qualified and complete successful rate of the iStent group, control group, POAG subgroup, and NTG subgroup. Qualified success was defined as reduction of at least 1 antiglaucoma agent with IOP less than 21 mmHg, and complete success was defined as medication-free with IOP less than 21 mmHg. (a) At 12 months follow-up, the iStent group showed a significantly higher qualified successful rate (68.2% vs. 14.7%, *p* value < 0.001) and complete successful rate (50.0% vs. 8.8%, *p* value = 0.001) than the control group. (b) At 18-month follow-up, the iStent group still showed a significantly higher qualified successful rate (73.3% vs. 16.7%, *p* value = 0.001) and complete successful rate (53.3% vs. 0%, *p* value = 0.002) than the control group. (c) At 12 months, the NTG-iStent subgroup showed the highest qualified successful rate (75%) and complete successful rate (75%). However, there is no significant difference between the NTG-iStent subgroup and POAG-iStent subgroup. (d) At 18 months, the NTG-iStent subgroup showed an even higher qualified successful rate (87.5%) and complete successful rate (87.5%). The complete successful rate was significantly higher than the POAG-iStent subgroup at 18-month follow-up (*p* value = 0.041).

The iStent group exhibited significantly higher qualified successful rates compared to the control group, with rates of 68.2% versus 14.7% at 12 months (*p* value < 0.001) and 73.3% versus 16.7% at 18 months (*p* value = 0.001). Similarly, the iStent group also demonstrated higher complete successful rates at both 12 months (50.0% vs. 8.8%, *p* value = 0.001) and 18 months (53.3% vs. 0%, *p* value = 0.002) compared to the control group (Figure 3(a) and 3(b)). Additionally, the iStent group showed better improvement in visual acuity than the control group, which was significant at 1, 3, 6, 9, and 12 months of follow-up (Supporting Figure 2, Supporting Table 6). The RNFL thickness, MD, and VFI also showed better outcome in the iStent group; however, the results were not significant (Supporting Figure 3).

### 6.4. POAG and NTG Subgroup Analysis

**Figure 4:**
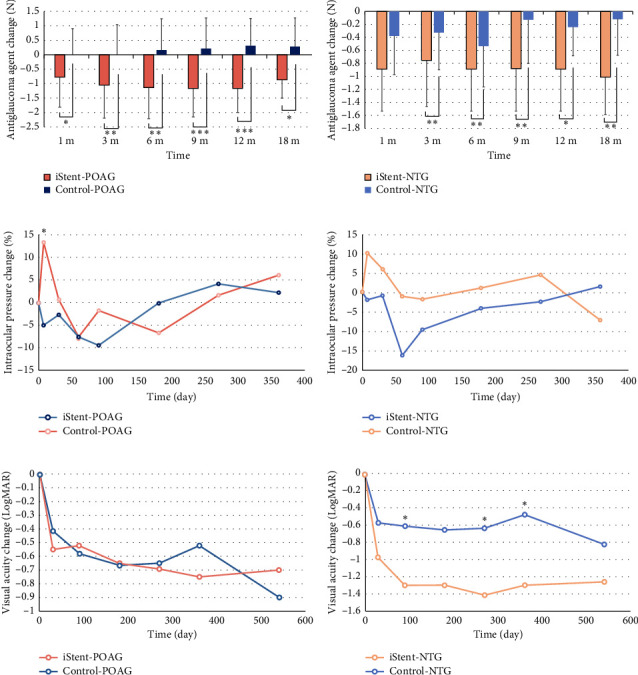
The antiglaucoma agent changes, intraocular pressure (IOP) changes, and visual acuity changes (LogMAR) in primary open-angle glaucoma (POAG) and normal-tension glaucoma (NTG) subgroup throughout the 18-month follow-up. (a) In POAG patients, the iStent group showed reduction of antiglaucoma agents after the surgery while the control group showed slightly increase of antiglaucoma agents. (b) In NTG patients, both the iStent group and control group showed a reduction of antiglaucoma agents after the surgery. In addition, the iStent group showed significantly better medication reduction capability than the control group. (c) In patients with POAG, the control group experienced an IOP surge 1 week after surgery (*p* value = 0.043), whereas both the iStent group and control group exhibited similar long-term IOP changes. (d) In the normal-tension glaucoma (NTG) subgroup, there were no significant differences in IOP changes between the iStent group and the control group. (e) In POAG patients, the iStent group and control group showed similar visual acuity improvement after the surgery. (f) In NTG patients, the iStent group showed more apparent visual acuity improvement than the control group. There were significant differences noted at 3 (−1.29 ± 0.65 in the iStent group and −0.60 ± 0.32 in the control group, *p* value = 0.020), 9 (−1.41 ± 0.72 in the iStent group and −0.63 ± 0.35 in the control group, *p* value = 0.016), and 12 (−1.29 ± 0.76 in the iStent group and −0.47 ± 0.47 in the control group, *p* value = 0.023) months follow-up.

In the subgroup analysis of patients with POAG and NTG, we noted a substantial decrease in the requirement for antiglaucoma medications following iStent implantation in both cohorts (refer to Figures 4(a), 4(b), and Supporting Table 7). Regarding IOP variations, the control group experienced an IOP surge at the 1-week follow-up (*p* value = 0.043) in the POAG subgroup, while no significant differences between the groups were seen in the NTG subgroup throughout the follow-up period (refer to Figures 4(c), 4(d), and Supporting Table 8). Additionally, the NTG-iStent group demonstrated significantly better visual acuity improvement compared to the NTG-control group at 3, 9, and 12 months, whereas no significant difference was observed in the POAG subgroups (Figures 4(e), 4(f), and Supporting Table 9). Furthermore, the iStent group achieved significantly higher qualified and complete success rates compared to the control group at 12 and 18 months in both POAG and NTG patients (Figures 3(c) and 3(d)). Notably, the NTG-iStent group had the highest qualified and complete success rates, significantly outperforming the POAG-iStent group (*p* = 0.041).

## 7. Discussion

In this case-control, single-surgeon study, we observed a better IOP control in patients receiving phaco-iStent compared with phacoemulsification alone. On average, patients in the iStent group had reduced one medication at 18-month follow-up without significant IOP rebound. The efficacy of phaco-iStent was similar for POAG and NTG, averagely reducing one medication. Nevertheless, the NTG had significantly higher complete surgical success rate than POAG (85.7% vs. 25.0%, *p* value = 0.041, Figure 3(d)).

In our study, we introduced a novel concept of estimating IOP without medication washout, which we termed as “ewIOP.” This approach can be easily calculated by multiplying the IOP by the mean IOP reduction (%) for each antiglaucoma drug. We assess the “ewIOP” instead of “washout IOP” owns two important advantages: 1. Do no harm to the participants. Patients do not have to bear the risk of IOP elevation during washout. 2. Easier to monitor serial IOP changes in the long term because researchers do not require repeated washout before data collection. Although individuals may respond differently to the same drug, and the additive effects of drugs also vary, it can be an alternative way to evaluate the impact of surgery on both IOP and medication changes without exposing patients to the risks of high-IOP-related injury during the washout period [17, 18]. However, the efficacy of glaucoma medications in reducing IOP may not align with expected outcomes, potentially leading to bias. Therefore, additional validation of this formula is warranted.

We employed generalized estimating equations to account for all potential confounders and assess the direct impact of iStent implantation on changes in the requirement for antiglaucoma medications and ewIOP reduction, both revealing a notable decrease in the iStent group compared to the control group (*β* = −0.8, SE = 0.165, *p* < 0.001 for antiglaucoma medication changes and *β* = −16.083, SE = 4.099, *p* < 0.001 for ewIOP). Our study showed similar results to previous reports that combining iStent implantation with phacoemulsification could reduce IOP and/or antiglaucoma agent use [10, 19–21]. While the direct comparison between studies may be arbitrary due to differing designs, backgrounds, surgeons, and indications for reintroducing antiglaucoma agents after the procedure, we encourage the use of ewIOP in future studies. This approach provides a comprehensive assessment of IOP and glaucoma medication changes simultaneously and can aid in the comparison between different studies. However, further studies are necessary to verify the correlation between the estimated washout IOP and true washout IOP.

The surgical success rate for the iStent group in our study was comparable (50% at 12 months and 53.3% at 18 months) to previous reports (range from 27.8% to 77%) [9, 19, 22–25]. Additionally, NTG eyes had a significantly higher surgical success rate (85.7% at 18 months) compared to POAG eyes, implying that phaco-iStent is more likely to fail in eyes with higher preoperative IOP. A previous study on bovine eyes has reported a decrease in outflow facility with increasing IOP. This finding suggested that elevated IOP may lead to aqueous plexus collapse and collector channel ostia blockage, further increasing outflow resistance [26]. This may explain why eyes with higher preoperative IOP had lower surgical success in the current study. The NTG patients may benefit more from phaco-iStent because of the relatively intact aqueous outflow system. To our knowledge, this study is one of the early studies comparing the effect of phaco-iStent with phaco alone in NTG patients, and further research is needed to explore the mechanism underlying this finding. Consistent with our findings, varying degrees of IOP reduction were reported in different types of open-angle glaucoma following glaucoma surgery. Tekcan et al. reported that phacoemulsification following trabeculotomy provided superior long-term IOP control and reduced the need for additional glaucoma surgeries in pseudoexfoliation glaucoma compared to POAG [27]. Another study showed that gonioscopy-assisted transluminal trabeculotomy achieved greater IOP reduction in pseudoexfoliative glaucoma within the first-year postsurgery compared to POAG [28]. Understanding differences in IOP response among different glaucoma subtypes could help clinicians develop optimal treatment strategies for individual patients.

This study is the first case-control study to report on the clinical outcome of phaco-iStent in a Taiwanese population, and one of the early studies to evaluate its efficacy in the NTG subgroup. Additionally, we introduced the concept of “estimated washout IOP” for the evaluation of IOP without medication washout. However, the study had several limitations, including a small sample size, a retrospective and nonrandomized design, a relatively short follow-up period, and the utilization of an ewIOP rather than the actual washout IOP. This approach may introduce bias due to variations in the effectiveness of IOP reduction among individual patients. Despite these limitations, the findings are still noteworthy and warrant further investigation in larger, prospective studies.

## 8. Conclusions

In conclusion, our study demonstrated the efficacy of phaco-iStent in reducing the need for antiglaucoma medication in patients with OAG, particularly benefiting those with NTG. At 18-month follow-up, patients in the iStent group could reduce one medication without experiencing IOP rebound. Additionally, 50% of OAG patients who underwent iStent-phaco achieved medication-free status. The NTG subgroup had the highest medication-free proportion, reaching up to 85.7% at 18 months. Our results suggest that patients with poor compliance, glaucoma drug intolerance, or NTG may benefit the most from phaco-iStent.

## Figures and Tables

**Table 1:** Patient characteristics.

Basic characteristics	iStent group	Control group	*p* value
	(*N* = 24)	(*N* = 47)	
OD/OS, *n*	15/9	24/23	0.360^a^
Age, mean ± sd (years)	68.71 ± 7.04	70.96 ± 11.17	0.142^c^
Sex, M/F	12/12	19/28	0.442^a^
Disease diagnosis, *n*	POAG: 16/NTG: 8	POAG: 28/NTG: 19	0.560^a^
Underlying disease			
Hypertension, *n* (%)	4 (16.7%)	7 (14.9%)	1.000^b^
Diabetes mellitus, *n* (%)	5 (20.8%)	15 (29.8%)	0.420^a^
Heart diseases, *n* (%)	2 (8.3%)	2 (4.3%)	0.599^b^
Baseline VA (LogMAR), mean ± sd	1.24 ± 0.54	1.13 ± 0.44	0.435^c^
Baseline IOP (mmHg), mean ± sd	18.83 ± 3.06	18.61 ± 4.24	0.547^c^
Baseline estimated washout IOP (mmHg), mean ± sd	22.26 ± 5.81	21.35 ± 6.08	0.548^d^
Baseline antiglaucoma agent use (*N*), mean ± sd	1.83 ± 1.20	1.36 ± 0.92	0.169^c^
Baseline disc OCT thickness (uM), mean ± sd	59.45 ± 21.04	65.39 ± 20.25	0.299^d^
Baseline MD (dB), mean ± sd	−13.17 ± 10.21	−12.64 ± 10.37	0.675^c^
Baseline VFI (%), mean ± sd	60.05 ± 35.70	65.06 ± 34.47	0.285^c^

Abbreviations: F, female; IOP, intraocular pressure; M, male; MD, mean deviation; NTG, normal-tension glaucoma; OCT, optical coherence tomography; OD, oculus dextrus; OS, oculus sinister; POAG, primary open-angle glaucoma; VA, visual acuity; VFI, visual field index.

^a^Chi-square test.

^b^Fisher's exact test.

^c^Mann–Whitney *U* test.

^d^Student's *t*-test.

**Table 2:** Generalized estimating equation (GEE) analysis of the correlation between iStent use and the reduction of antiglaucoma medication in 18-month follow-up.

	*β*	SE	*p* value
iStent insertion	−0.8	0.165	<0.001^∗∗∗^
Eyesite (OD/OS)	−0.224	0.154	0.146
Age	−0.043	0.015	0.003^∗∗^
Sex	0.046	0.190	0.810
Disease diagnosis (POAG/NTG)	0.389	0.198	0.049
Underlying diseases			
DM	0.184	0.190	0.331
HTN	−0.087	0.265	0.743
CAD	0.465	0.319	0.144
Baseline VA (LogMAR)	−0.270	0.248	0.277
Baseline IOP	−0.008	0.024	0.721
Baseline antiglaucoma medication use	−0.451	0.087	< 0.001^∗∗∗^
Baseline disc OCT thickness	0.017	0.006	0.002^∗∗^
Baseline MD	0.157	0.046	0.001^∗∗^
Baseline VFI	−0.059	0.014	< 0.001^∗∗∗^
Axial length	0.007	0.061	0.903
Anterior chamber depth	−0.718	0.239	0.003

Abbreviations: IOP, intraocular pressure; MD, mean deviation; NTG, normal-tension glaucoma; OCT, optical coherence tomography; OD, oculus dextrus; OS, oculus sinister; POAG, primary open-angle glaucoma; VA, visual acuity; VFI, visual field index.

⁣^∗^*p* < 0.05.

⁣^∗∗^*p* < 0.01.

⁣^∗∗∗^*p* < 0.001.

## Data Availability

The data used to support the findings of this study are available from the corresponding author upon request.

## Supporting Information

Supporting Information 1 Supporting Figure 1. The estimated washout IOP changes (%) in the iStent group and control group throughout the 18-month follow-up.

Supporting Information 2 Supporting Figure 2. The visual acuity changes (LogMAR) in the iStent group and control group throughout the 18-month follow-up.

Supporting Information 3 Supporting Figure 3. The disc optical coherence tomography (OCT) thickness change, visual field mean deviation (MD) change, and visual field visual field index (VFI) change after the 12-month follow-up.

Supporting Information 4 Supporting Table 1. Estimating washout intraocular pressure weighting of each antiglaucoma agent.

Supporting Information 5 Supporting Table 2. IOP changes in the iStent group and control group.

Supporting Information 6 Supporting Table 3. Changes in the number of antiglaucoma medications in the iStent and control groups.

Supporting Information 7 Supporting Table 4. Change in estimated washout intraocular pressure (ewIOP) in iStent and Control Groups.

Supporting Information 8 Supporting Table 5. Generalized estimating equation (GEE) analysis of the correlation between iStent use and the estimated washout IOP change in 18-month follow-up.

Supporting Information 9 Supporting Table 6. Change in visual acuity (LogMAR) in iStent and control groups.

Supporting Information 10 Supporting Table 7. Changes in the number of antiglaucoma medications in POAG and NTG subgroup analysis.

Supporting Information 11 Supporting Table 8. Change in intraocular pressure (IOP) in POAG and NTG subgroup analyses.

Supporting Information 12 Supporting Table 9. Change in visual acuity (LogMAR) in POAG and NTG subgroup analysis.
