# Supplementary material for: Comparison of Phacoemulsification Alone and With Trabecular Microbypass Stent in Primary Open-Angle Glaucoma and Normal-Tension Glaucoma: An 18-Month Outcome Study
Source: J Ophthalmol. 2024 Nov 7;2024:4034215. doi: 10.1155/2024/4034215 (PMC11563717; doi:10.1155/2024/4034215)
Supplement: Supporting Information 1 — Supporting Figure 1. The estimated washout IOP changes (%) in the iStent group and control group throughout the 18-month follow-up. [file 4034215.f1.pdf]

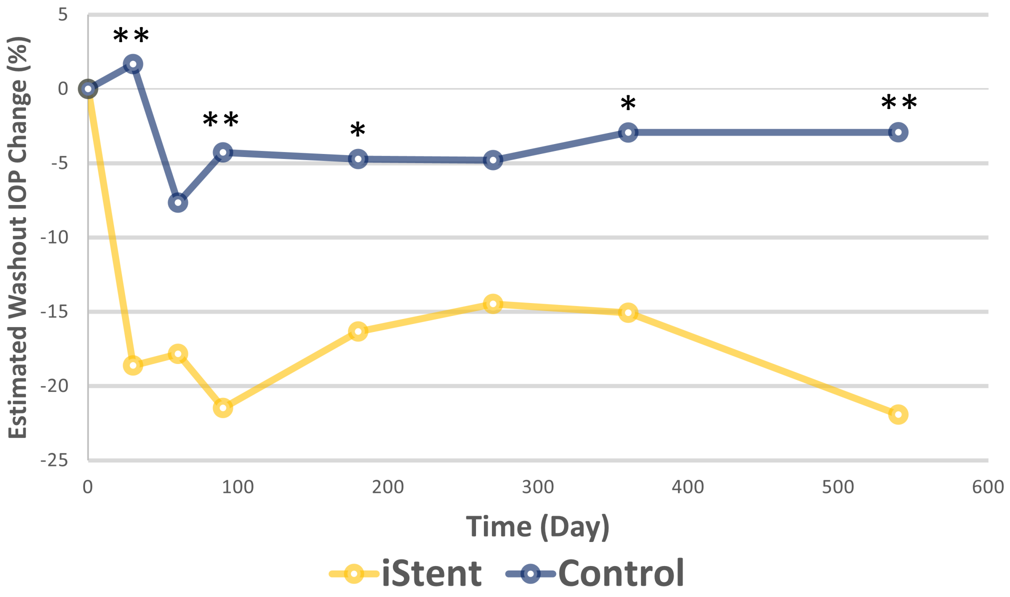


**Supplemental Figure 1. The estimated washout IOP changes (%) in the iStent group and control group throughout the 18 months follow-up.** The average reduction of estimated washout IOP in iStent group was greater than the control group throughout the 18 months follow-up and showed significant differences at 1 month (-18.62 ± 29.58 % in the iStent group and 1.66 ± 30.07 % in control group, P value = 0.009), 3 months (-21.48 ± 14.36 % in the iStent group and -4.27 ± 29.84 % in the control group, P value = 0.009), 6 months (-16.34 ± 15.50% in iStent group and -4.73 ± 23.90% in control group, P value = 0.023), 12 months (-15.06 ± 13.82% in iStent group and -2.92 ± 29.76% in control group, P value = 0.045), and 18 months (-21.92 ± 14.75% in iStent group and -2.92 ± 21.99% in control group, P value = 0.005). The

estimated washout IOP was calculated by multiplying the IOP and the mean IOP reduction effect of each antiglaucoma agent.
