# Supplementary material for: Comparison of Phacoemulsification Alone and With Trabecular Microbypass Stent in Primary Open-Angle Glaucoma and Normal-Tension Glaucoma: An 18-Month Outcome Study
Source: J Ophthalmol. 2024 Nov 7;2024:4034215. doi: 10.1155/2024/4034215 (PMC11563717; doi:10.1155/2024/4034215)
Supplement: Supporting Information 2 — Supporting Figure 2. The visual acuity changes (LogMAR) in the iStent group and control group throughout the 18-month follow-up. [file 4034215.f2.pdf]

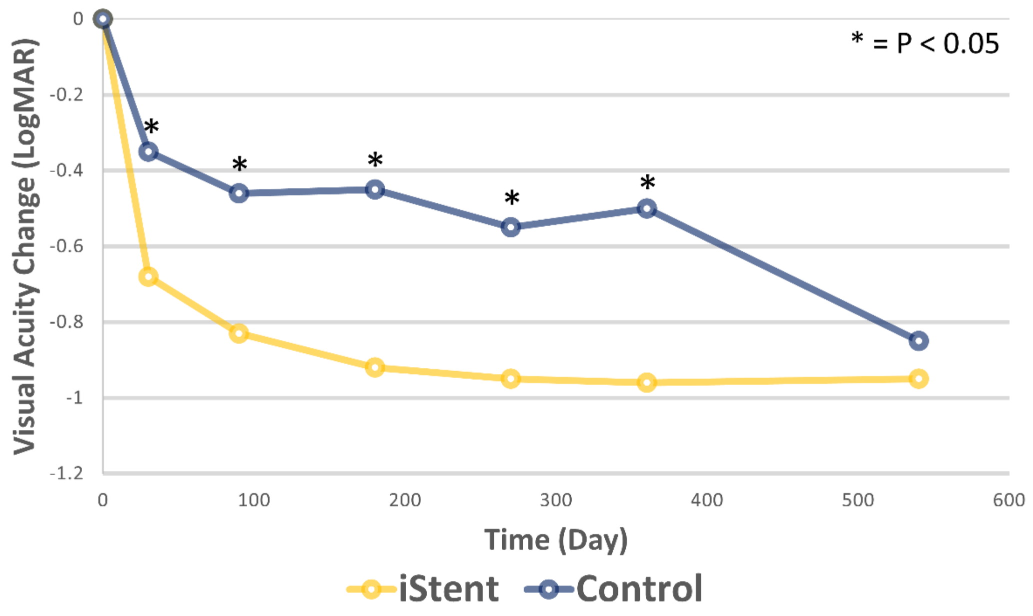

Supplemental Figure 2. The visual acuity changes (LogMAR) in iStent group and control group throughout the 18 months follow up. The iStent group showed better visual acuity improvement throughout the 18 months follow up. The results were significant different at 1 (-0.68 ± 0.61 in iStent group and -0.35± 0.41 in control group, *P* value = 0.039), 3 (-0.83 ± 0.75 in iStent group and -0.46 ± 0.30 in control group, *P* value = 0.042), 6 (-0.92 ± 0.68 in iStent group and -0.45 ± 0.53 in control group, *P* value = 0.024), 9 (-0.95 ± 0.70 in iStent group and -0.55 ± 0.31 in control group, *P* value = 0.035), 12 (-0.96 ± 0.63 in iStent group and -0.50 ± 0.56 in control group, *P* value= 0.017) months follow up.
