# Supplementary material for: Comparison of Phacoemulsification Alone and With Trabecular Microbypass Stent in Primary Open-Angle Glaucoma and Normal-Tension Glaucoma: An 18-Month Outcome Study
Source: J Ophthalmol. 2024 Nov 7;2024:4034215. doi: 10.1155/2024/4034215 (PMC11563717; doi:10.1155/2024/4034215)
Supplement: Supporting Information 3 — Supporting Figure 3. The disc optical coherence tomography (OCT) thickness change, visual field mean deviation (MD) change, and visual field visual field index (VFI) change after the 12-month follow-up. [file 4034215.f3.pdf]

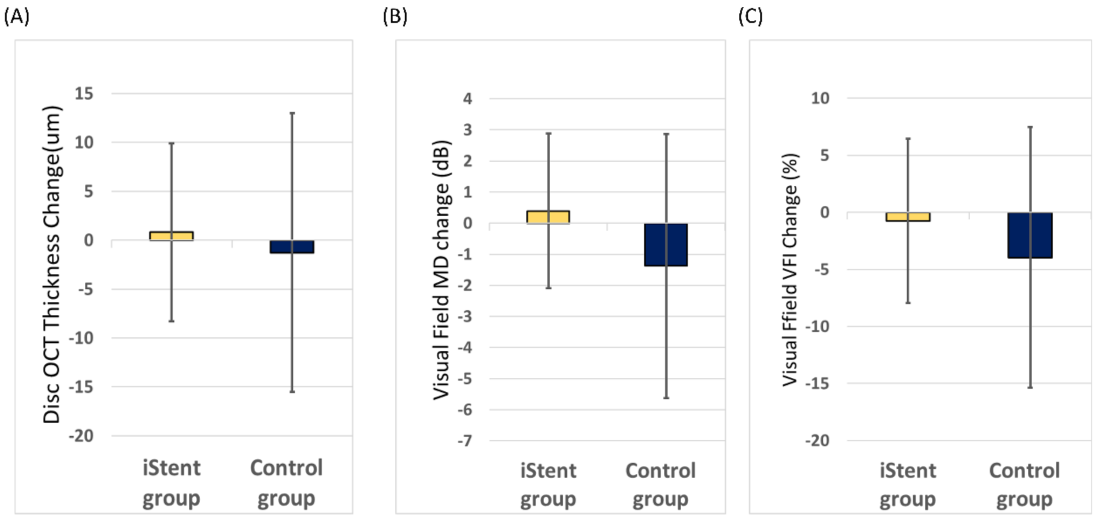

Supplemental Figure 3. The disc optical coherence tomography (OCT) thickness change, visual field mean deviation (MD) change, and visual field visual field index (VFI) change after 12 months follow up. (A) The disc OCT thickness was slightly increased (0.82 ± 9.1 um) in iStent group and slightly decreased (-1.28 ± 14.25 um) in control group after 12 months of the surgery. However, there's no significant differences between the two groups. (B) The MD of visual field was slightly increased (0.39 ± 2.49 dB) in iStent group and slightly decreased (-1.38 ± 4.24 dB) in control group after 12 months of the surgery. However, there's no significant differences between the two groups. (C) The VFI showed slightly decrease in both iStent group (-0.75 ± 7.19 %) and control group after 12 months (-3.95 ± 11.41 %) of the surgery and there's no significant differences.
