# Supplementary material for: Comparison of Phacoemulsification Alone and With Trabecular Microbypass Stent in Primary Open-Angle Glaucoma and Normal-Tension Glaucoma: An 18-Month Outcome Study
Source: J Ophthalmol. 2024 Nov 7;2024:4034215. doi: 10.1155/2024/4034215 (PMC11563717; doi:10.1155/2024/4034215)
Supplement: Supporting Information 4 — Supporting Table 1. Estimating washout intraocular pressure weighting of each antiglaucoma agent. [file 4034215.f4.pdf]

Supplemental Table 1. Estimating washout intraocular pressure weighting of each antiglaucoma agents

Prostaglandin analogues	
Latanoprost	1.285
Travoprost	1.285
Bimatoprost	1.3
Tafluprost	1.29
Latanoprostene bunod	1.31
β-Adrenergic antagonist (β-blockers)	
Timolol maleate	1.25
Timolol hemihydrate	1.25
Levobunolol	1.25
Metipranolol	1.25
Betaxolol	1.175
α_2-Adrenergic agonists	
Apraclonidine hydrochloride	1.25
Brimonidine tartrate 0.2%	1.25
Brimonidine tartrate in Purite 0.1%	1.25
Carbonic anhydrase inhibitors	
Acetazolamide (Oral)	1.175
Acetazolamide (Parenteral)	1.175
Methazolamide	1.175
Dorzolamide (Topical)	1.175
Brinzolamide (Topical)	1.175
Cholinergic agonist	
Pilocarpine HCl	1.2
Anticholinesterase agent	
Echothiophate iodide	1.2
Rho kinase inhibitors	
Netarsudil	1.205
Fixed combinations	
Timolol/brinzolamide	1.275
Timolol/dorzolamide	1.275
Timolol/travoprost	1.275
Timolol/bimatoprost	1.275
Timolol/brimonidine tartrate	1.275
Brinmonidine/brinzolamide	1.31

The data was referenced by Table 7-1 of the BCSC 2020-2021 series: Section 10 – Glaucoma
