# Supplementary material for: Comparison of Phacoemulsification Alone and With Trabecular Microbypass Stent in Primary Open-Angle Glaucoma and Normal-Tension Glaucoma: An 18-Month Outcome Study
Source: J Ophthalmol. 2024 Nov 7;2024:4034215. doi: 10.1155/2024/4034215 (PMC11563717; doi:10.1155/2024/4034215)
Supplement: Supporting Information 5 — Supporting Table 2. IOP changes in the iStent group and control group. [file 4034215.f5.pdf]

Supplemental Table 2. IOP changes in iStent group and control group

| | iStent group | Control group | |
|---|---|---|---|
| Case number | N = 24 | N = 47 | *P* value |
| IOP Day0 (baseline) | 15.55 ± 2.47 mmHg | 15.50 ± 3.28 mmHg | 0.946[a] |
| IOP change 1 month | -0.58 ± 4.75 mmHg | 0.31 ± 3.53 mmHg | 0.379[a] |
| IOP change 2 months | -1.45 ± 3.29 mmHg | -1.15 ± 2.75 mmHg | 0.531[b] |
| IOP change 3 months | -1.60 ± 2.21 mmHg | -0.66 ± 3.47 mmHg | 0.242[a] |
| IOP change 6 months | -0.26 ± 2.02 mmHg | -0.94 ± 3.18 mmHg | 0.248[b] |
| IOP change 9 months | 0.13 ± 2.56 mmHg | -0.81 ± 3.39 mmHg | 0.274[a] |
| IOP change 12 months | 0.21 ± 2.80 mmHg | -0.71 ± 3.79 mmHg | 0.333[a] |
| IOP change 18 months | -1.11 ± 2.84 mmHg | -0.89 ± 3.12 mmHg | 0.832[a] |

The results were analyzed by Student's t test for the normally-distributed data and Mann–Whitney U test for the non-normally distributed data; [a]: Student's t test; [b]: Mann–Whitney U test

IOP: intraocular pressure;
