# Supplementary material for: Comparison of Phacoemulsification Alone and With Trabecular Microbypass Stent in Primary Open-Angle Glaucoma and Normal-Tension Glaucoma: An 18-Month Outcome Study
Source: J Ophthalmol. 2024 Nov 7;2024:4034215. doi: 10.1155/2024/4034215 (PMC11563717; doi:10.1155/2024/4034215)
Supplement: Supporting Information 6 — Supporting Table 3. Changes in the number of antiglaucoma medications in the iStent and control groups. [file 4034215.f6.pdf]

Supplemental Table 3. Changes in the Number of Antiglaucoma Medications in the iStent and Control Groups

	iStent group	Control group	
Case number	N = 24	N = 47	*P* value
Number of antiglaucoma agent use Day0 (baseline)	1.83 ± 1.20	1.36 ± 0.92	0.169
Number of antiglaucoma agent change 1 month	-0.75 ± 0.94	-0.13± 0.61	0.003**
Number of antiglaucoma agent change 3 months	-1.00 ± 0.98	-0.02 ± 0.89	<0.001***
Number of antiglaucoma agent change 6 months	-1.04 ± 0.95	0.08 ± 0.14	<0.001***
Number of antiglaucoma agent change 9 months	-1.08 ± 0.88	0.05 ± 0.91	<0.001***
Number of antiglaucoma agent change 12 months	-1.05 ± 0.79	0.03 ± 0.80	<0.001***
Number of antiglaucoma agent change 18 months	-0.93 ± 0.59	0.05 ± 0.80	<0.001***

The results were analyzed by Mann–Whitney U test for all the non-normally distributed data.

* for $p < 0.05$, ** for $p < 0.01$, *** for $p < 0.001$
