# Supplementary material for: Comparison of Phacoemulsification Alone and With Trabecular Microbypass Stent in Primary Open-Angle Glaucoma and Normal-Tension Glaucoma: An 18-Month Outcome Study
Source: J Ophthalmol. 2024 Nov 7;2024:4034215. doi: 10.1155/2024/4034215 (PMC11563717; doi:10.1155/2024/4034215)
Supplement: Supporting Information 7 — Supporting Table 4. Change in estimated washout intraocular pressure (ewIOP) in iStent and Control Groups. [file 4034215.f7.pdf]

Supplemental Table 4. Change in Estimated Washout Intraocular Pressure (ewIOP) in iStent and Control Groups

	iStent group	Control group	
Case number	N = 24	N = 47	*P* value
Estimated washout IOP Day0 (baseline, mmHg)	22.26 ± 5.81	21.35 ± 6.08	0.548
Changes of Estimated washout IOP 1 month (%)	-18.62 ± 29.58	1.66 ± 30.07	0.009**
Changes of Estimated washout IOP 2 month (%)	-17.84 ± 23.98	-7.65± 26.89	0.168
Changes of Estimated washout IOP 3 month (%)	-21.48 ± 14.36	-4.27 ± 29.84	0.002**
Changes of Estimated washout IOP 6 month (%)	-16.34 ± 15.50	-4.73 ± 23.90	0.023*
Changes of Estimated washout IOP 9 month (%)	-14.47 ± 19.18	-4.79 ± 23.68	0.12
Changes of Estimated washout IOP 12 month (%)	-15.06 ± 13.82	-2.92 ± 29.76	0.045*
Changes of Estimated washout IOP 18 month (%)	-21.92 ± 14.75	-2.92 ± 21.99	0.005**

The results were analyzed by Student's t test for all the normally distributed data.

IOP: intraocular pressure * for p < 0.05, ** for p < 0.01, *** for p<0.001
