# Supplementary material for: Comparison of Phacoemulsification Alone and With Trabecular Microbypass Stent in Primary Open-Angle Glaucoma and Normal-Tension Glaucoma: An 18-Month Outcome Study
Source: J Ophthalmol. 2024 Nov 7;2024:4034215. doi: 10.1155/2024/4034215 (PMC11563717; doi:10.1155/2024/4034215)
Supplement: Supporting Information 8 — Supporting Table 5. Generalized estimating equation (GEE) analysis of the correlation between iStent use and the estimated washout IOP change in 18-month follow-up. [file 4034215.f8.pdf]

Supplemental Table 5. Generalized estimating equation (GEE) analysis of the correlation between iStent use and the estimated washout IOP change in 18 months follow up

	β	SE	*P* value
iStent insertion	-16.083	4.099	<0.001***
Eyesite (OD)	0.196	3.965	0.961
Age	-0.324	0.335	0.334
Gender (M)	10.894	5.211	0.037*
Disease diagnosis (NTG)	6.930	5.206	0.183
Underlying diseases			
DM	3.574	4.645	0.442
HTN	-4.468	6.565	0.496
CAD	-1.836	8.017	0.819
Baseline VA(LogMAR)	6.051	5.925	0.307
Baseline IOP	0.052	0.743	0.944
Baseline estimated washout IOP	-1.824	0.584	0.002**
Baseline antiglucoma agent use	-1.043	2.961	0.725
Baseline disc OCT thickness	-0.052	0.138	0.707
Baseline MD	1.028	1.187	0.387
Baseline VFI	-0.397	0.360	0.270

IOP: intraocular pressure; MD: mean deviation; NTG: normal tension glaucoma; OCT: Optical Coherence Tomography; OD: oculus dextrus; OS: oculus sinister; POAG: primary open-angle glaucoma; VA: visual acuity; VFI: visual field index

*: p < 0.05; **: p < 0.01; ***: p < 0.001
