# Supplementary material for: Comparison of Phacoemulsification Alone and With Trabecular Microbypass Stent in Primary Open-Angle Glaucoma and Normal-Tension Glaucoma: An 18-Month Outcome Study
Source: J Ophthalmol. 2024 Nov 7;2024:4034215. doi: 10.1155/2024/4034215 (PMC11563717; doi:10.1155/2024/4034215)
Supplement: Supporting Information 9 — Supporting Table 6. Change in visual acuity (LogMAR) in iStent and control groups. [file 4034215.f9.pdf]

Supplemental Table 6. Change in Visual Acuity (LogMAR) in iStent and Control Groups

	iStent group	Control group	
Case number	N = 24	N = 47	*P* value
VA (LogMAR) Day0 (baseline)	1.24 ± 0.54	1.13 ± 0.44	0.365
VA (LogMAR) change 1 month	-0.68 ± 0.61	-0.35± 0.41	0.039*
VA (LogMAR) change 3 months	-0.83 ± 0.75	-0.46 ± 0.30	0.042*
VA (LogMAR) change 6 months	-0.92 ± 0.68	-0.45 ± 0.53	0.024*
VA (LogMAR) change 9 months	-0.95 ± 0.70	-0.55 ± 0.31	0.035*
VA (LogMAR) change 12 months	-0.96 ± 0.63	-0.50 ± 0.56	0.017*
VA (LogMAR) change 18 months	-0.95 ± 0.69	-0.85 ± 0.41	0.643

The results were analyzed by Student's t test for all the normally distributed data.

VA: visual acuity * for $p < 0.05$, ** for $p < 0.01$, *** for $p<0.001$
