# Supplementary material for: Comparison of Phacoemulsification Alone and With Trabecular Microbypass Stent in Primary Open-Angle Glaucoma and Normal-Tension Glaucoma: An 18-Month Outcome Study
Source: J Ophthalmol. 2024 Nov 7;2024:4034215. doi: 10.1155/2024/4034215 (PMC11563717; doi:10.1155/2024/4034215)
Supplement: Supporting Information 10 — Supporting Table 7. Changes in the number of antiglaucoma medications in POAG and NTG subgroup analysis. [file 4034215.f10.pdf]

Supplemental Table 7. Changes in the Number of Antiglaucoma Medications in POAG and NTG Subgroup Analysis

Case number	POAG subgroup			NTG subgroup		
	iStent group (N = 16)	Control group (N = 28)	P value	iStent group (N = 8)	Control group (N=19)	P value
Number of antiglaucoma agent use Day0 (baseline)	2.19 ± 1.33	1.39 ± 1.10	0.062	1.13 ± 0.35	1.32 ± 0.58	0.585
Number of antiglaucoma agent change 1 month	-0.75 ± 1.06	0.00 ± 0.92	0.012*	-0.75 ± 0.71	-0.32 ± 0.58	0.147
Number of antiglaucoma agent change 3 months	-1.06 ± 1.12	0.00 ± 1.06	0.002**	-0.88 ± 0.64	-0.05 ± 0.62	0.009**
Number of antiglaucoma agent change 6 months	-1.12 ± 1.09	0.19 ± 1.08	0.001**	-0.88 ± 0.64	-0.05 ± 0.62	0.009**
Number of antiglaucoma agent change 9 months	-1.19 ± 0.98	0.21 ± 1.08	<0.001***	-0.88 ± 0.64	-0.11 ± 0.68	0.022*
Number of antiglaucoma agent change 12 months	-1.14 ± 0.86	0.29 ± 0.99	<0.001***	-0.88 ± 0.64	-0.24 ± 0.44	0.031*
Number of antiglaucoma agent change 18 months	-0.88 ± 0.64	0.25 ± 1.04	0.021*	-1.00 ± 0.58	-0.10 ± 0.57	0.014*

The results were analyzed by Mann–Whitney U test for all the non-normally distributed data.

* for p < 0.05, ** for p < 0.01, *** for p<0.001
