# Supplementary material for: Comparison of Phacoemulsification Alone and With Trabecular Microbypass Stent in Primary Open-Angle Glaucoma and Normal-Tension Glaucoma: An 18-Month Outcome Study
Source: J Ophthalmol. 2024 Nov 7;2024:4034215. doi: 10.1155/2024/4034215 (PMC11563717; doi:10.1155/2024/4034215)
Supplement: Supporting Information 11 — Supporting Table 8. Change in intraocular pressure (IOP) in POAG and NTG subgroup analyses. [file 4034215.f11.pdf]

Supplemental Table 8. Change in Intraocular Pressure (IOP) in POAG and NTG Subgroup Analysis

	POAG subgroup (N = 44)			NTG subgroup (N = 27)		
Case number	iStent group (N = 16)	Control group (N = 28)	P value	iStent group (N = 8)	Control group (N=19)	P value
Estimated washout IOP Day0 (baseline, mmHg)	16.06 ± 2.21	16.76 ± 3.04	0.423[a]	14.54 ± 2.79	13.64 ± 2.74	0.444[a]
Changes of Estimated washout IOP 1 week (%)	-4.89 ± 20.91	13.27 ± 27.35	0.043*[a]	-1.87 ± 41.89	10.02 ± 27.13	0.477
Changes of Estimated washout IOP 1 month (%)	-2.66 ± 21.51	0.73 ± 22.62	0.660[a]	-0.67 ± 48.60	6.05 ± 24.24	0.685[a]
Changes of Estimated washout IOP 2 month (%)	-7.53 ± 10.75	-7.89 ± 18.56	0.953[a]	-16.13 ± 22.63	-1.02 ± 15.97	0.126[a]
Changes of Estimated washout IOP 3 month (%)	-9.41 ± 14.60	-1.71 ± 21.60	0.261[a]	-9.72 ± 11.40	-1.94 ± 21.00	0.381[a]
Changes of Estimated washout IOP 6 month (%)	-0.13 ± 13.80	-6.64 ± 19.22	0.307[a]	-4.20 ± 11.86	-1.17 ± 25.67	0.592[a]
Changes of Estimated washout IOP 9 month (%)	4.17 ± 19.86	1.69 ± 21.47	0.767[a]	-2.48 ± 7.28	4.55 ± 21.55	0.423[a]
Changes of Estimated washout IOP 12 month (%)	2.28 ± 17.68	6.04 ± 21.22	0.627[a]	1.48 ± 18.98	-7.05 ± 30.13	0.497[a]

The results were analyzed by Student's t test for the normally-distributed data and Mann–Whitney U test for the non-normally distributed data; [a]: Student's t test; [b]: Mann–Whitney U test

IOP: intraocular pressure; * for $p < 0.05$, ** for $p < 0.01$, *** for $p<0.001$
