# Supplementary material for: Comparison of Phacoemulsification Alone and With Trabecular Microbypass Stent in Primary Open-Angle Glaucoma and Normal-Tension Glaucoma: An 18-Month Outcome Study
Source: J Ophthalmol. 2024 Nov 7;2024:4034215. doi: 10.1155/2024/4034215 (PMC11563717; doi:10.1155/2024/4034215)
Supplement: Supporting Information 12 — Supporting Table 9. Change in visual acuity (LogMAR) in POAG and NTG subgroup analysis. [file 4034215.f12.pdf]

Supplemental Table 9. Change in Visual Acuity (LogMAR) in POAG and NTG Subgroup Analysis

Case number	POAG subgroup			NTG subgroup		
	iStent group (N = 16)	Control group (N = 28)	P value	iStent group (N = 8)	Control group (N=19)	P value
VA (LogMAR) Day0 (baseline)	1.22 ± 0.50	1.22 ± 0.48	0.965[a]	1.47 ± 0.64	0.99 ± 0.33	0.09[b]
VA (LogMAR) change 1 month	-0.55 ± 0.59	-0.41 ± 0.64	0.494 [a]	-0.96 ± 0.65	-0.56 ± 0.31	0.133 [a]
VA (LogMAR) change 3 months	-0.52 ± 0.68	-0.58 ± 0.53	0.771 [a]	-1.29 ± 0.65	-0.60 ± 0.32	0.020 [a,*]
VA (LogMAR) change 6 months	-0.65 ± 0.48	-0.66 ± 0.64	0.951 [a]	-1.29 ± 0.76	-0.64 ± 0.44	0.053[a]
VA (LogMAR) change 9 months	-0.69 ± 0.60	-0.65 ± 0.50	0.82 [a]	-1.41 ± 0.72	-0.63 ± 0.35	0.016[b,*]
VA (LogMAR) change 12 months	-0.75 ± 0.43	-0.52 ± 0.63	0.321 [a]	-1.29 ± 0.76	-0.47 ± 0.47	0.023[a,*]
VA (LogMAR) change 18 months	-0.70 ± 0.55	-0.90 ± 0.57	0.484 [a]	-1.25 ± 0.76	-0.82 ± 0.26	0.162[a]

The results were analyzed by Student's t test for the normally-distributed data and Mann–Whitney U test for the non-normally distributed data; [a]: Student's t test; [b]: Mann–Whitney U test

* for p < 0.05, ** for p < 0.01, *** for p<0.001
